# The molecular mechanism of leaf margin fission in *Solanum nigrum* revealed by combined PPI network and WGCNA and functional validation of *SnNAC90*

**DOI:** 10.3389/fpls.2025.1706416

**Published:** 2025-11-18

**Authors:** Hongquan Shen, Man Miao, Zhoumin Cha, Zexi Fan, Qihang Cai, Zhenghai Sun, Liping Li

**Affiliations:** 1 Yunnan International Joint R&D Center for Integrated Utilization of Ornamental Grass/International Technological Cooperation Base of Highly Effective Economic Forestry cultivating of Yunnan Province/South and Southeast Asia Joint R&D Center of Economic Forest Full Industry Chain of Yunnan Province, College of Landscape and Horticulture of Southwest Forestry University, Kunming, China; 2 College of Geography and Ecotourism, Southwest Forestry University, Kunming, China

**Keywords:** *Solanum nigrum*, transcriptome sequencing, lobe, WGCNA, functional verification

## Abstract

**Introduction:**

*Solanum nigrum*, a member of the Solanaceae family, holds significant importance in various aspects, including edible and medicinal uses, ecological management, and environmental landscaping. The leaf edges of *S. nigrum* exhibit 4–5 significant serrations, and the splitting of these leaf margins plays a crucial role in the plant’s adaptation to environmental shifts.

**Methods:**

In this study, we systematically analyzed the molecular mechanism of leaf margin fission in *S. nigrum* by combining RNA sequencing, weighted gene co-expression network analysis, and overexpression transgenic technology using leaves from five growth stages of *S. nigrum* during the flowering period as materials.

**Results:**

Transcriptome analysis revealed that 30,311 differentially expressed genes were activated from leaf bud to mature leaf, and these genes were significantly enriched in metabolic pathways related to signal transduction and glycosynthesis. Differential genes were hierarchically clustered into 13 modules. The correlations of these modules with different growth stages of *S. nigrum* leaves, as well as the number and depth of leaf notches were analyzed. It was found that the turquoise expression module (ME1) was significantly and positively correlated with the leaf bud stage (r = 0.94, p < 0.01), while negatively correlated with the number and depth of leaf notches. Three highly connected hub genes were identified from network interactions analysis of genes related to the leaf differentiation pathway in Module 1. From the intersection of the turquoise module and the 2 clusters screened by temporal analysis, the core gene (*SnNAC90*) for leaf margin fission in lobelia was identified. The regulatory role of the *SnNAC90* gene in tobacco leaves was preliminarily verified by transgenic technology.

**Discussion:**

It was hypothesized that it might positively regulate leaf margin fission in *S. nigrum*. Preliminary speculation on its regulatory role in *S. nigrum* leaves. This study, for the first time, revealed the regulatory mechanism of leaf margin division in *S. nigrum*, providing a theoretical basis for enriching its germplasm resources and serving as a reference basis for cultivating leaf plants.

## 1 Introduction

The leaf is the primary organ responsible for photosynthesis and energy conversion in plants. Leaf traits are important for nutrient cycling and primary productivity (Wright et al., 2004). Plant leaf margin fission is an important phenotype in plant adaptation to environmental changes. In herbaceous plants, deeply notched leaf plants are taller than shallowly notched leaf plants, leaf margin notching gives plants a greater advantage in their ability to grow longitudinally, and deeply cleft leaf plants capture light energy more quickly and compete better for limited light sources (Semchenko and Zobel, 2007). In addition, studies have shown that plants with deeply lobed leaves can dissipate heat quickly by improving air flow and reducing heat transfer distances, so plants with notched leaves can withstand higher temperatures than plants with unnotched leaves (Song et al., 2021; Tserej and Feeley, 2021). The notching along the edges of leaves impacts the drought resistance of plants, where those with deep lobes show greater drought tolerance and are more suited to water shortages compared to those with shallow lobes (Tserej and Feeley, 2021). Leaf shape is closely related to genetic information and external environmental factors. Existing theories of leaf morphogenesis are mainly based on model plants such as *Arabidopsis thaliana* (Du et al., 2018).

NAC proteins represent a significant group of plant-specific transcription factors, extensively present in terrestrial plants. NAC genes are abundant in plants, with more than 100 genes in *Arabidopsis thaliana* (Singh et al., 2021). Up until now, research has been conducted on only a small portion of NAC proteins, yet these proteins play a role in numerous functions, such as defense mechanisms, non-living stress reactions, and growth processes (Puranik et al., 2012; Nuruzzaman et al., 2013; Shao et al., 2015; Yuan et al., 2019; Dong et al., 2024; Fuertes-Aguilar and Matilla, 2024; Xiong et al., 2025). Hegedus et al. proposed that NAC proteins play a role in biological stress reactions by triggering them in potatoes (*Solanum tuberosum*); for instance, the StNAC gene (Hegedus et al., 2003; Singh et al., 2013; Xu et al., 2014; Yue et al., 2021) and the triggering of various kale-type oilseed rape (*Brassica napus*) NAC genes via insect feeding and fungal infections (Hegedus et al., 2003). Some of these genes are also expressed by abiotic stresses such as trauma, cold shock, and dehydration (Puranik et al., 2012), and transgenic plants that overexpress three different NAC genes (*AtNAC019*, *AtNAC055*, and *AtNAC072*) have been reported to significantly increase drought tolerance (Jensen et al., 2010). Aida et al. observed the fusion of CUC1-*CUC2* and a double mutant cotyledon in *A. thaliana*, the removal of embryonic SAM, and altered flower development during bud regeneration in the healing mutant tissue (Gonçalves et al., 2015). CUC proteins synergize with the KNOX transcription factor family to fine-tune the depth and number of lobular cleavages by activating the cyclin gene *CYCB1*, which promotes cell proliferation in the lobular meristematic tissue region while inhibiting genes associated with differentiation to remain undifferentiated (Tsuda and Hake, 2015; Wei et al., 2022).

*Solanum nigrum*, belonging to the Solanaceae family and indigenous to Southeast Asia, has expanded extensively across Europe, Asia, and the Americas, particularly in tropical and subtropical regions (Chen et al., 2022; Wei et al., 2022). *S. nigrum*, commonly referred to as “Yelahu,” “Yehaijiao,” “Heixingxing,” “Heitiantian,” “Kukui,” “Kucai,” “Heidoudou,” and “Yesanzi,” is extensively found across various regions of China, typically thriving in wild areas, fields, and diverse habitats (Chen et al., 2022). *S. nigrum* exhibits robust energy and a remarkable ability to adapt to environmental changes, allowing it to grow in various soil types (Wang et al., 2024). *S. nigrum* is also of high culinary and medicinal value and has a long history of edible and medicinal uses in its area of origin (Zhang et al., 2023). *S. nigrum* is capable of efficiently absorbing cadmium from its soil surroundings, thereby diminishing the cadmium levels in the soil (Wang et al., 2024; Zheng et al., 2024). In summary, current studies on *S. nigrum* have primarily focused on medical aspects and soil management.

Weighted Gene Correlation Network Analysis (WGCNA) is a new network modeling method that improves simple correlation networks based on easy-to-understand statistical methods (Maertens et al., 2018). Utilizing this method, one can identify clusters of jointly expressed genes and their association with external characteristics and locate crucial central genes; it is extensively applied in recognizing genes that are co-expressed due to stress and the arrangement of cell walls in cotton (You et al., 2016), common gene expression of *Arabidopsis thaliana* and rice on salt stress (Razzaque et al., 2019; Mohamed et al, n.d.), and genes that are co-expressed as a reaction to living stressors in *A. thaliana* (Burks et al., 2022). In this study, the leaf transcriptomes of *S. nigrum* were sequenced at five different stages of flowering (leaf bud, lobule, mid-leaf, large leaf, and mature leaf), and genes that regulate leaf margin fission of *S. nigrum* were screened using the WGCNA method. The application of overexpression transgenic methods to introduce the specific gene into tobacco (*Nicotiana benthamiana*) serves as an initial step to confirm the gene’s function, offering fresh perspectives for examining both the traditional and novel aspects of leaf cleavage control in *S. nigrum*, as well as for researching plant morphological evolution.

## 2 Materials and methods

### 2.1 Plant sample planting

Seeds of *S. nigrum* gathered from Yunnan’s field were immersed in a solution of 1% hydrogen peroxide for five minutes, then promptly rinsed 3–5 times with sterile water. The seeds were then soaked in warm water at a temperature of 30°C for a quarter of an hour to activate them. The activated seeds were then placed on sterilized wet filter paper in a thermostat at 28°C for germination. To keep the filter paper moist, water it twice a day with pure water during the germination stage. Post-germination, the seeds were relocated into 50-hole standard trays using a soil blend of humus and perlite in a 3:1 ratio. After the transplant, the tray was placed in a greenhouse at the Southwest Forestry University Botanic Garden (25.06626° N, 102.76824° E, 1960 m above sea level) and watered regularly at a constant temperature of 28°C. When the seedlings grow to 3–4 cotyledons, we transferred them to a 20 cm diameter pot with the same soil as in a cavity tray.

### 2.2 Sample gathering and processing

Foliage from *S. nigrum’s* five growth phases (buds, small, medium, large, and mature leaves) during its blooming phase was gathered from Southwest Forestry University’s greenhouse (**Supplementary Figure S1**), ensuring three biological duplicates per stage. Quantitative metrics for black slave blades include the number of notches, notch depth, blade area and notch area, where area is measured by the grid method. Each sample was numbered SnL1-1, SnL1-2, SnL1-3, SnL2-1, SnL2-2, SnL2-3, SnL3-1, SnL3-2, SnL3-3, SnL4-1, SnL4-2, SnL4-3, SnL5-1, SnL5-2, and SnL5-3, and each sample contained not less than 200 mg. The leaves were cleaned before gathering. After washing, the samples were transferred into 50 mL freezing tubes that had been pre-cooled and treated with liquid nitrogen, and they were then instantly frozen in liquid nitrogen. After that, they were brought back to the laboratory and kept in a refrigerator at minus 80 degrees Celsius before being shipped to Tsingke Biotechnology Co., Ltd. (Kunming Branch) for sequencing and library construction.

### 2.3 Library construction, quality control, and sequencing

(1) Constructing the library: total RNA was extracted using the TRIzol technique, and its purity (OD260/280 1.8–2.0) and integrity (RIN ≥ 7.0) were verified using the NanoDrop test and agarose gel electrophoresis. After enrichment by oligo (dT) magnetic beads, mRNA was cleaved by divalent cations at 94°C into fragments 200–300 bp in length. Following the synthesis of the first strand of cDNA using six-base random primers (random hexamers), the second strand was synthesized using DNA polymerase I, RNase H, and dNTPs. The cDNA library was then created using end repair, A-tail addition, sequencing junction ligation, and PCR amplification. (2) Quality control: Qubit 2.0 was utilized for initial quantification, followed by qPCR quantification (≥2 nM) to confirm the library’s quality, and the Agilent 2100 Bioanalyzer to identify fragment distribution (300–500 bp). (3) Sequencing: After cluster generation for qualified libraries, double-end sequencing was performed using the Illumina NovaSeq 6000 platform. The sequencing method is sequencing by side sequencing (SBS), and the fluorescence signal is converted into sequence reads by a standard protocol. Raw data were output in FASTQ format.

### 2.4 Data quality control

In the analysis of reference-free transcriptome data, the quality control firstly assessed the raw sequencing data (fastq format) obtained from the sequencing platform and detected the base quality distribution, GC content, and splice contamination using the FastQC tool and then eliminated the low-quality bases (reads with the proportion of N greater than 10%; the number of bases with Q ≤ 10 accounted for more than 50% of the whole read) and junction sequences and retained clean reads ≥ 50 bp in length. The data complexity was finally assessed by KmerGenie analysis to ensure that the percentage of valid sequences was >90% and that there was no significant batch effect, providing high-quality data for subsequent transcript assembly.

### 2.5 Sequence comparison with sequenced data

In this study, spliced transcripts were used as comparative reference sequences, and transcriptome splicing was performed using software called Trinity (Haas et al., 2013). Clarity reads were patched together using Trinity, a patchwork of transcripts containing a large number of shorter transcripts, a patchwork of Python scripts extracting the longest transcripts as non-redundant gene sets, and the final non-redundant gene sequences obtained and stored in FASTA format. Clear readings from each sample were compared with reference sequences using Hisat2 software (Haas et al., 2013; Guo et al., 2022).

### 2.6 Quantification of gene expression

Clean reads were reposted to the unigene sequence via Hisat2 software, which counted the reads counts for each unigene and generated the raw count matrix. The expression levels of genes and transcripts were measured using stringtie software by counting the amount of mapped reads on the genes, which was based on the negative binomial distribution (Jiang and Wong, 2009) model. The length of the transcripts and the number of mapped reads in the samples were normalized so that the number of fragments accurately reflected the degree of transcript expression. To gauge the level of transcript or gene expression, use FPKM (Florea et al., 2013) (fragments per kilobase of transcript per million fragments mapped). The expected number of transcript sequence fragments per length per million base pairs that have been sequenced is known as FPKM. One of the most popular techniques for determining gene expression levels also considers the impact of sequencing depth and gene length.

### 2.7 Differential gene screening

To determine whether genes are differently expressed between two biological circumstances, DESeq (Altschul, 1997) can be used for differential expression analysis between sample groups for physiologically duplicated samples. The number of differently expressed genes and the experimental criteria can be used to modify the multiplicity of gene changes during differential gene detection. In this experiment, Fold Change ≥ 2 and FDR < 0.01 were used as screening criteria. Fold change indicates the ratio of gene expression between the experimental and control groups. The False Discovery Rate (FDR) is obtained by modifying the meaning of the differential *p*-value (*p*-value). Since differential expression analysis of LncRNA sequencing can lead to errors and impact mRNA expression detection, as well as possible false positives, the significance *p*-value (*p*-value in the original hypothesis test modified using the recognized Benjamini–Hochberg correction method) ultimately uses FDR as a key indicator for differential expression gene screening.

### 2.8 Differential gene enrichment analysis

The unreferenced transcriptome’s differential gene enrichment analysis began with a search for unigenes with coding potential using CPC2. Then, using BLAST (Altschul, 1997) software, the codable unigenes were compared with public databases like Nr, Swiss-Prot, KOG/COG, GO, and KEGG to obtain the functional annotation information of the differential genes. GO functional enrichment analysis (including biological processes, cellular components, and molecular functions) and KEGG pathway enrichment analysis for differential unigenes were performed using clusterProfiler based on the annotation results. The hypergeometric test was used to determine the significance of enrichment, and the significantly enriched GO entries and KEGG pathways were screened using the threshold value of adjusted *p*-value < 0.05. The KEGG database integrates information from genomes, chemical compounds, and biochemical systems to comprehensively examine the metabolic pathways of gene products in cells and their roles. Utilizing this database facilitates the analysis of gene and expression data as a comprehensive network.

### 2.9 Process and parameters of WGCNA analysis

WGCNA looks for highly relevant gene clusters (modules) by constructing gene-gene co-expression networks and linking this information to the traits being measured for use in the search for relevant candidate genes. Based on the theory that genes with similar expression profiles may have close functional links or pathways, this method provides a systematic analysis for studying functional clustering of expression profiles. Firstly, differentially expressed genes (DEGs) with similar expression trends were clustered into a module by hierarchical clustering. The modules most relevant to the phenotype were then identified through correlation analysis between the modules and the phenotype. In this study, the β soft threshold for hierarchical clustering was set to 6, with a scale-free R^2^ > 0.8, and the minimum number of modular genes was 30. Modules with |r|>0.8 and P ≤ 0.01 were considered highly correlated in the analysis of module phenotype correlations across individual growth stages.

### 2.10 Acquisition of transgenic tobacco

Transgenic tobacco plants were produced using the leaf disk method (Horsch et al., 1985), and the tobacco used in this study was cultivated in an experimental greenhouse at Southwest Forestry University. We chose healthy 4–5-week-old tobacco plants, picked fully puffed young leaves (3rd–5th leaf position 3–5) from sterile tables, and disinfected them. Sterilization involved immersing in 75% ethanol for a duration of 10 s, followed by three rapid rinses with sterile water, an 8 min immersion in 0.1% HgCl_2_, and ultimately, five washes with sterile water. The sterilized tobacco was cut into 2 cm cubes and set aside. Agrobacterium tumefaciens GV3101 was inoculated with the overexpression vector containing the target gene. Single colonies were selected and inoculated into LB liquid medium containing the appropriate antibiotics. The colonies were then cultured at 28 °C with 200 rpm shaking until OD600 = 0.8–1.0. The colonies were then harvested by centrifugation and resuscitated with an equivalent volume of liquid MS medium. After 10 min of soaking in reconstituted Agrobacterium solution, the cut tobacco leaves were placed on co-culture medium for 48 h of dark incubation. The Agrobacterium liquid on the leaf surface was then wiped off with sterile filter paper. Subsequently transferred to screening medium (2.25 mg/L 6-BA, 0.3 mg/L NAA, 20 mg/L thaumatin, and 400 mg/L cephalosporin) for resistance screening; the medium was changed every 2 weeks. After the resistant buds grew, they were inoculated into strong bud medium (2.25 mg/L 6-BA, 0.3 mg/L NAA, 20 mg/L thaumatin, and 400 mg/L cephalosporin) for strong bud culture. Well-developed seedlings were then selected for rooting (rooting medium containing 20 mg/L thaumatin and 400 mg/L cephalosporin) culture. When the seedlings formed a good root system, they were removed and washed with water to remove the root agar, and then transplanted to the greenhouse for culture.

## 3 Results

### 3.1 Sequencing data analysis

**Figure 1:**
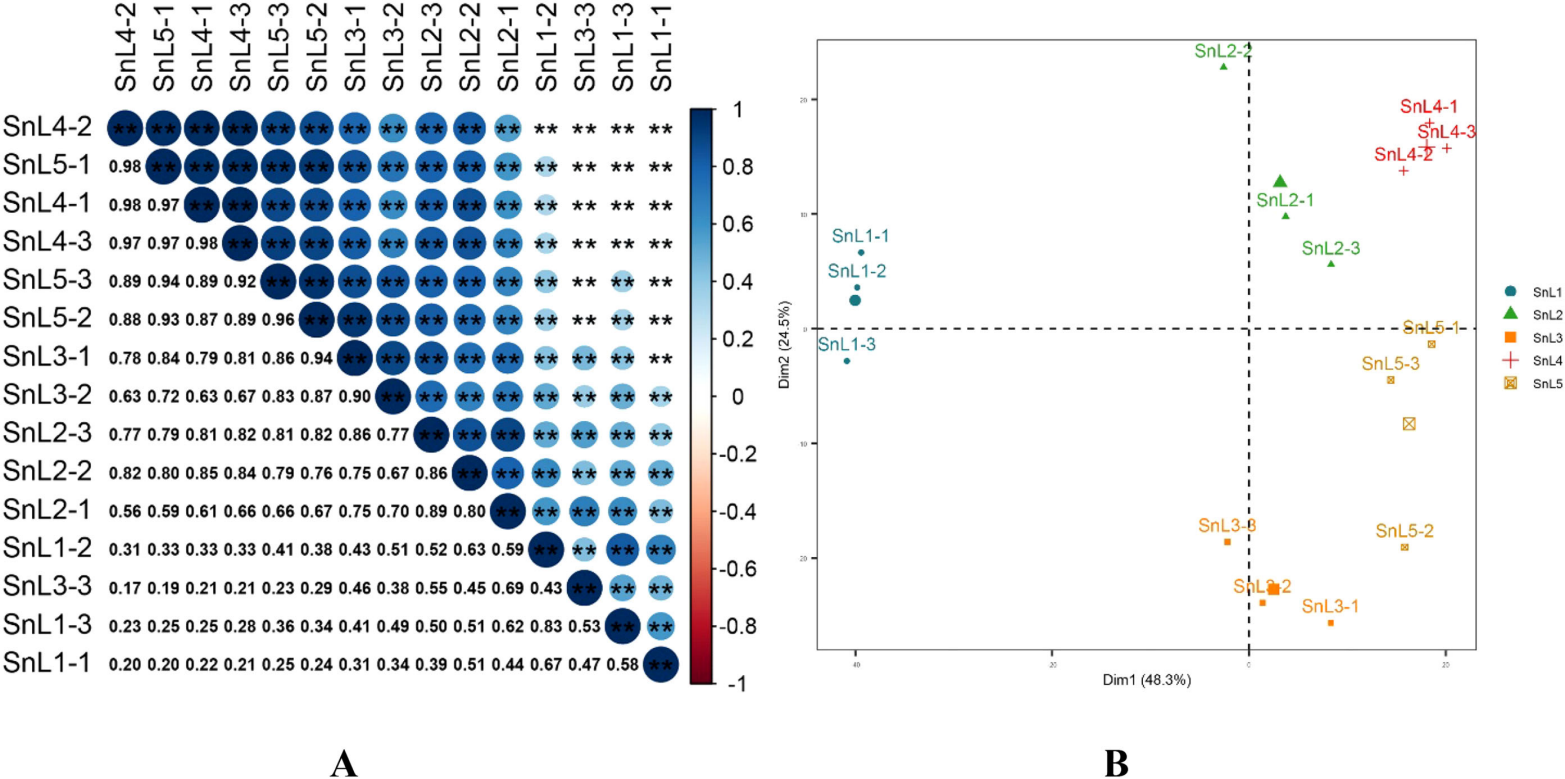
Sample correlation and PCA analyses reveal differences between different growth stages of *Solanum nigrum*. **(A)** RNA sequencing (RNA-seq) profiles of leaf margin fission phenotypes based on leaves at five growth stages (leaf bud, leaflet, mesophyll, leaflet, and maturity) during the flowering stage of *Solanum nigrum* were analyzed for correlation between samples using Pearson correlation coefficients: the size and color intensity of bubbles in the diagram represent correlation coefficients; larger bubbles with darker colors indicate higher correlations, while smaller bubbles with lighter colors indicate lower correlations. **(B)** mapping for reducing dimensionality: Conducting principal component analysis (PCA) on sequencing samples using variance decomposition (Dim1: 48.3%, Dim2: 24.5%). "*" indicates the significance of the correlation between samples (p), where two "*" denote p < 0.01.

In this study, 53,175,099 reads were generated using 15 sample leaves based on five growth stages during the flowering period of *S. nigrum*. An average of 53,175,098 Clean reads were obtained per library after quality control and filtration, with sequenced quality values Q20 > 98% and Q30 > 97%. The raw data generated by the sequencing platform was filtered using Trimmomatic 0.32 software. The clean reads obtained after filtering were then compared with the non-redundant gene sets. The comparison results (**Table 1**) showed that the comparison rate of reads that could be compared to the gene set ranged from 79.88 to 86.99%, and the percentage of reads that were compared to the unique location of the transcript ranged from 32.96 to 45.91%. To detect the reproducibility of the samples, correlation analysis was performed on the sequenced samples. The correlation heatmap (**Figure 1A**) showed a Pearson correlation coefficient of more than 0.80 between the three samples from the same growth stage, indicating good biological replication. The results of the principal component analysis showed that the three biological replicates from the same growth stage clustered together, and the three samples from the leaf bud stage were farther away from the samples from the other growth stages, suggesting that the leaf bud stage was more different from the other growth stages (**Figure 1B**).

**Table 1:** Results of clean reads versus spliced transcripts.

Samples ID	Total Reads	Unmapped Reads (%)	Mapped Reads (%)	Secondary Alignments (%)	Unique Alignments (%)
SnL1-1	60,369,806	10,540,838 (17.46)	49,828,968 (82.54)	24,500,414 (40.58)	25,328,554 (41.96)
SnL1-2	55,712,224	9,308,691 (16.71)	46,403,533 (83.29)	22,875,458 (41.06)	23,528,075 (42.23)
SnL1-3	53,693,568	9,704,025 (18.07)	43,989,543 (81.93)	21,827,740 (40.65)	22,161,803 (41.27)
SnL2-1	46,134,026	7,803,441 (16.91)	38,330,585 (83.09)	18,972,643 (41.13)	19,357,942 (41.96)
SnL2-2	52,940,572	9,424,306 (17.8)	43,516,266 (82.2)	23,799,223 (44.95)	19,717,043 (37.24)
SnL2-3	48,491,896	7,209,636 (14.87)	41,282,260 (85.13)	19,974,879 (41.19)	21,307,381 (43.94)
SnL3-1	56,553,428	7,647,721 (13.52)	48,905,707 (86.48)	23,703,653 (41.91)	25,202,054 (44.56)
SnL3-2	43,565,172	6,517,206(14.96)	37,047,966 (85.04)	17,926,267 (41.15)	19,121,699 (43.89)
SnL3-3	58,997,708	9,561,812 (16.21)	49,435,896 (83.79)	24,196,722 (41.01)	25,239,174 (42.78)
SnL4-1	54,698,346	7,723,744 (14.12)	46,974,602 (85.88)	23,295,360 (42.59)	23,679,242 (43.29)
SnL4-2	47,612,096	7,289,057 (15.31)	40,323,039 (84.69)	20,093,002 (42.2)	20,230,037 (42.49)
SnL4-3	49,140,250	6,632,074 (13.5)	42,508,176 (86.5)	21,746,633 (44.25)	20,761,543 (42.25)
SnL5-1	49,345,340	7,445,463 (15.09)	41,899,877 (84.91)	20,522,724 (41.59)	21,377,153 (43.32)
SnL5-2	50,079,918	7,604,637 (15.19)	42,475,281 (84.81)	20,932,035 (41.8)	21,543,246 (43.02)
SnL5-3	52,200,868	8,603,312 (16.48)	43,597,556 (83.52)	21,472,031 (41.13)	22,125,525 (42.39)

Sample ID refers to the number of samples; total reads denote the count of clean reads on a single-ended basis; mapped reads indicate the quantity of reads aligned with the spliced transcript and the proportion of clean reads.

### 3.2 Differentially expressed genes analysis

**Figure 2:**
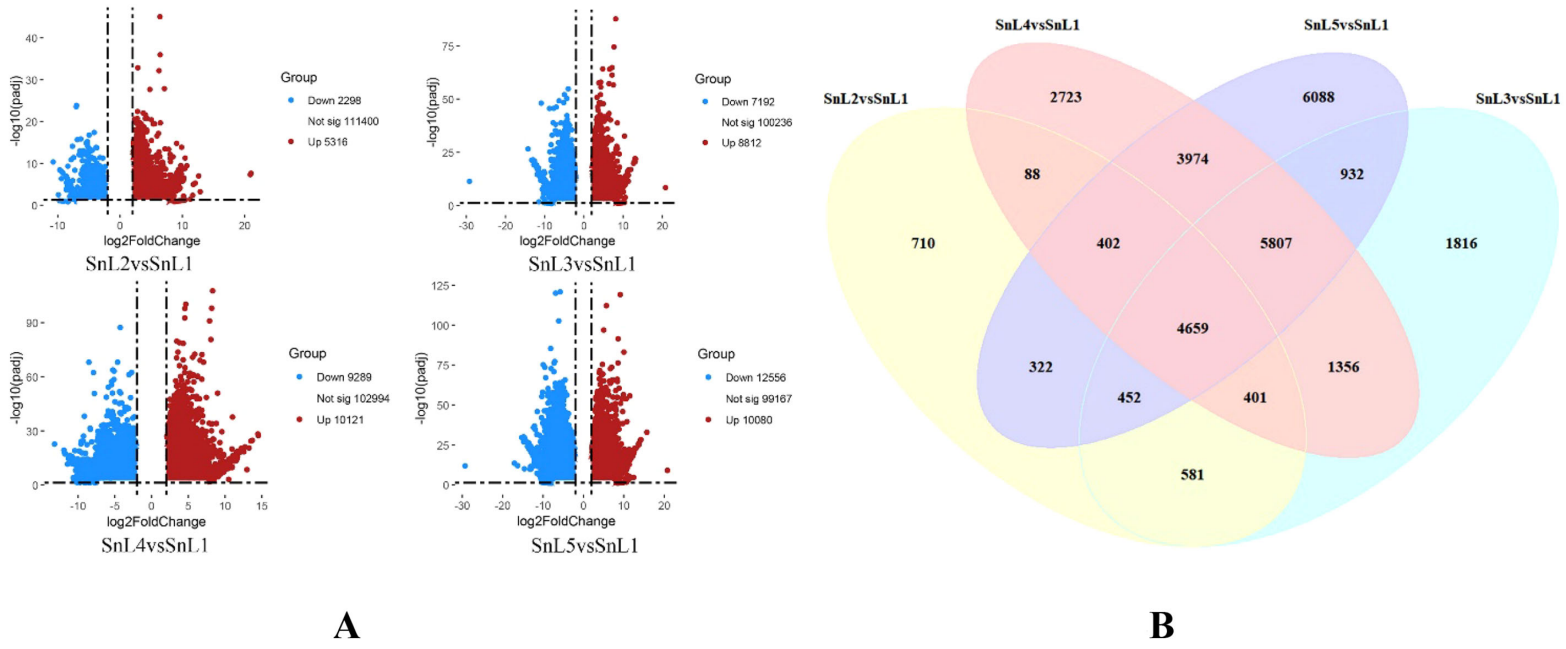
Variations in *Solanum nigrum*’s lobe cleavage gene expression by stage. Defined by |log2(fold change)| ≥ 2, FDR < 0.05, expressed genes (DEGs) are identified. **(A)** Number of DEGs in *Solanum nigrum* that are up- and down-regulated from leaf buds to mature leaves; **(B)** count of common DEGs in *Solanum nigrum*, ranging from leaf buds to fully developed leaves.

Measurements of *S. nigrum* leaves showed that the number of notches gradually increased and stabilized at the mid-leaf stage. The notch depth increased from 2 mm to 11 mm while the blade area expanded from 22.7 mm² to 2297.9 mm². Correspondingly, the notch area and notch-to-blade-area ratios exhibited a gradual and consistent upward trend (**Supplementary Table S1**). To unravel the molecular mechanisms behind leaf margin fission in *S. nigrum*, we analyzed transcriptional sequencing of *S. nigrum* leaves at five different growth stages during flowering (leaf buds, small leaves, medium leaves, large leaves, and mature leaves), and a total of 138,054 expressed genes were detected. The leaf bud stage (SnL1) was used as CK, and the differentially expressed genes were screened by thresholding (FDR < 0.05, |log2FC| > 2), and a total of 30,311 differentially expressed genes were screened; among them, 7614 DEGs (5316 up-regulated/2298 down-regulated) were screened in the leaflet stage (SnL2) compared to the leaf bud stage (SnL1), and 16,004 differential genes (8812 up-regulated/7192 down-regulated) were found in the midleaf stage (SnL3). There were 19,410 differential genes (10,121 up-regulated/9289 down-regulated) and 22,636 differential genes (10,080 up-regulated/12,556 down-regulated) at the large leaf (SnL4) and mature leaf stages (SnL5), respectively (**Figure 2A**). The number of differential genes at the above stages shows a gradual increase in the number of differential genes as the morphology of *S. nigrum* leaves is built up. The degree of leaf margin fission in *S. nigrum* is progressively deeper as the leaf develops and is phenotypically stable. Variation in the number of differential genes explains variation in leaf margins in *S. nigrum*. Venn diagram analysis revealed a total of 4659 differentially expressed genes across the four growth phases; these genes are predicted to have an important regulatory function in the process of leaf margin fission in *S. nigrum* (**Figure 2B**).

### 3.3 GO and KEGG analysis of common differentially expressed genes

**Figure 3:**
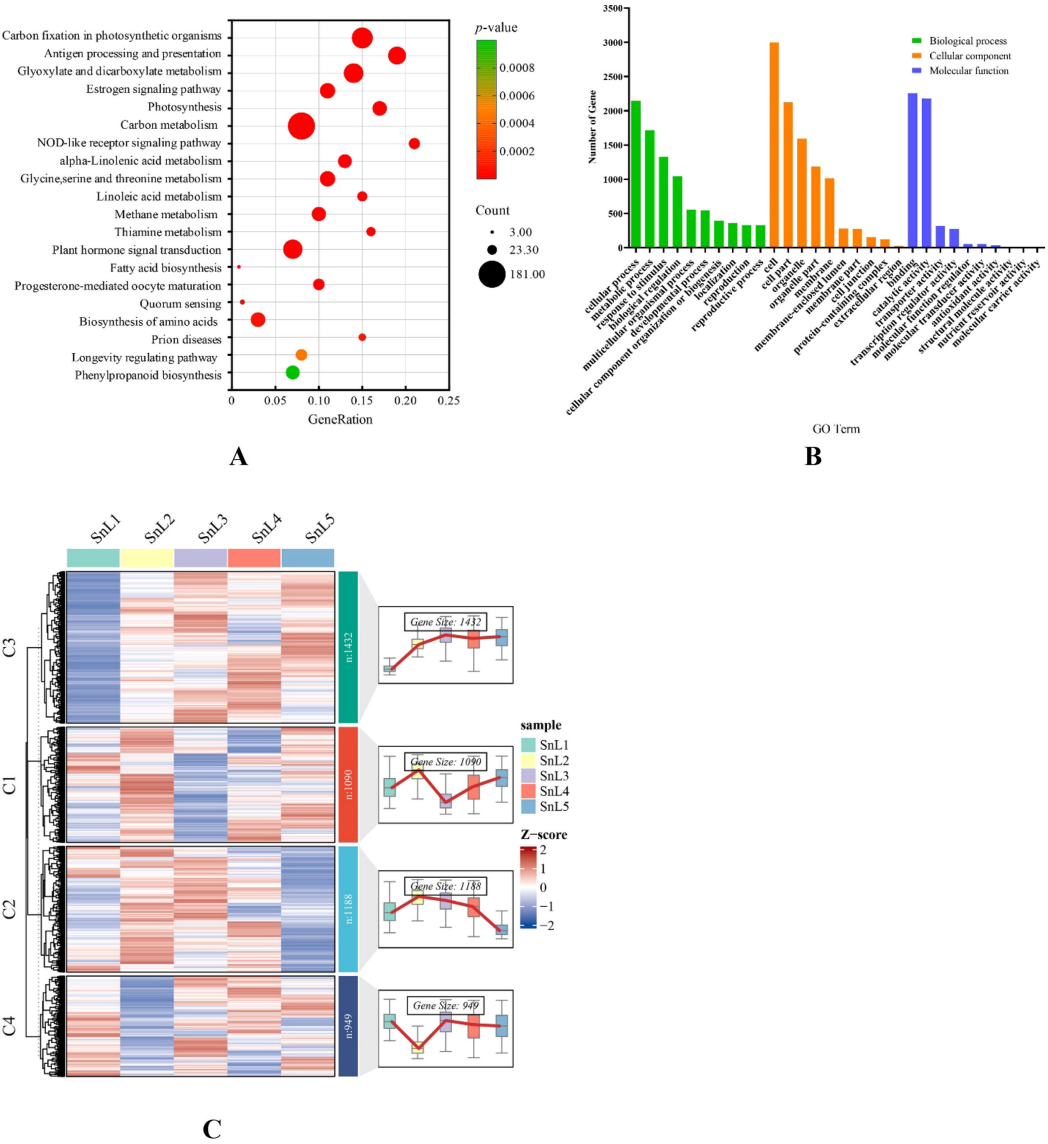
Enrichment analysis and temporal expression analysis of common differential genes. **(A)** top 10 terms for Biological Process (BP), Molecular Function (MF), and Cellular Component (CC) in a Gene Ontology (GO) enrichment analysis of common DEGs; **(B)** analysis of the KEGG pathway enrichment of common DEGs; **(C)** temporal analysis of common differential genes at five stages of leaf growth in *Solanum nigrum*: on the left is a heat map of clustering of common differential genes, and on the right is the expression trend of shared differential genes at five stages of leaf growth in *Solanum nigrum*.

KEGG and GO enrichment analysis of common differentially expressed genes was performed to explore the regulatory mechanism of leaf margin fission in *S. nigrum*. **Figure 3A** shows the results of GO enrichment in biological processes, which include cellular operations, metabolic processes, and responses to stimuli, among others. In cellular components, these common differential genes are mostly involved in the establishment of organelles and membranes. The molecular functions are mainly binding, catalytic activity, transporter activity, and transcriptional regulatory activity. The KEGG enrichment results (**Figure 3B**) revealed that these common differential genes were primarily enriched in carbon fixation in photosynthetic organisms, antigen processing and presentation, glyoxylate and dicarboxylate metabolism, the estrogen signaling pathway, photosynthesis, and carbon metabolism. By clustering the temporal expression patterns (**Figure 3C**), DEG was classified into four characteristic modules; some of the genes in cluster 1 and cluster 3 were highly expressed at the leaf bud stage. Cluster 1 genes had the lowest expression at the leaflet stage, but some genes showed high expression at the middle, large, and mature leaf stages, while cluster 3 genes had the lowest expression at the middle leaf stage, and some genes showed high expression at the large and mature leaf stages. It is noteworthy that cluster 2 and cluster 4 genes showed opposite expression profiles, with the cluster 2 gene being lowly expressed at the leaf bud stage. The expression of the cluster 2 gene gradually increased with the gradual development of the leaves, and its expression was relatively highest at the maturation stage. The cluster 4 gene showed high expression at the leaflet stage and the lowest expression at the mature leaf stage. In transcriptome sequencing, the expression of leaf cleavage-related genes decreases or increases progressively with the morphogenesis of plant leaves. The changes in the expression of cluster 2 and cluster 4 genes were consistent with the above pattern, and the cluster 2 and cluster 4 genes were analyzed in focus.

### 3.4 GO and KEGG analysis of genes in clusters 2 and 4

**Figure 4:**
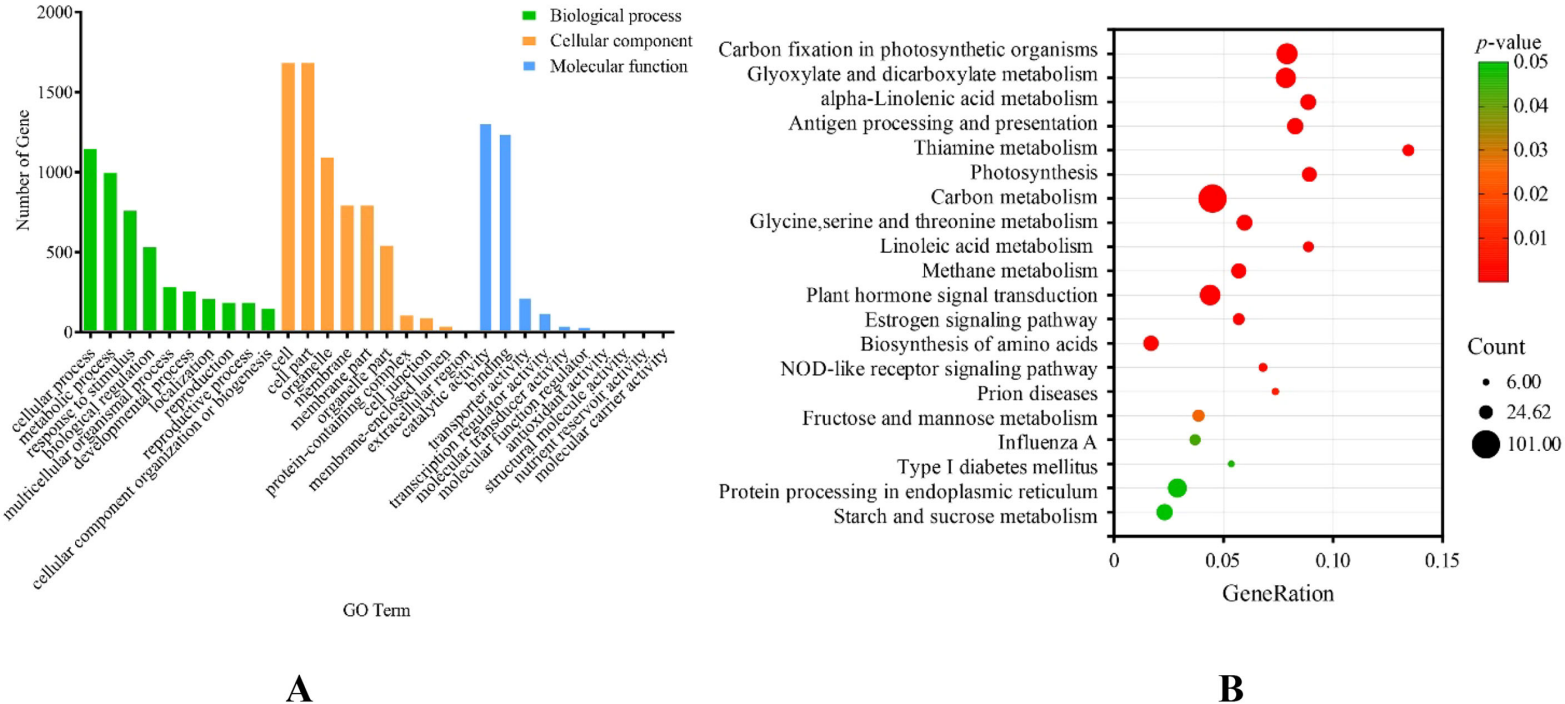
Enrichment analysis of cluster 2 and cluster 4 genes. **(A)** Analysis of Gene Ontology (GO) enrichment in genes of clusters 2 and 4, showing the top 10 terms in Biological Process (BP), Molecular Function (MF), and Cellular Component (CC); **(B)** KEGG pathway enrichment analysis of cluster 2 and cluster 4 genes.

The GO enrichment results (**Figure 4A**) demonstrate that the biological processes are mostly involved in cellular processes, metabolic processes, responses to stimuli, and developmental processes (cell differentiation/organ development/tissue morphogenesis), among others. The molecular functions are primarily enriched for binding (DNA/RNA/protein binding), catalytic activity, transporter activity, transcription regulator activity, molecular function regulator activity, and molecular transducer activity. In the molecular components, the main participants are the cell, cell parts, organelle, membrane, protein-containing complex, etc. According to KEGG enrichment analysis (**Figure 4B**), these genes are primarily involved in carbon fixation pathways in photosynthetic organisms, glyoxylate and dicarboxylate metabolism, alpha-linolenic acid metabolism, antigen processing and presentation, thiamine metabolism, photosynthesis, carbon metabolism, glycine, serine, and threonine metabolism, and so on. From the results of the above analysis, it was hypothesized that the mechanism of leaf margin fission formation in *S. nigrum* was stimulated by the external environment, which caused cell differentiation in the plant body through signal transduction to eventually form the *S. nigrum* leaf margin notched phenotype. Combined with previous studies, plant leaf cleavage phenotypes are associated with phenylpropane metabolism, starch and sucrose metabolism, terpene synthesis, signaling, and transporter activity, with a focus on genes enriched in the above pathways.

### 3.5 Transcription factor analysis

**Figure 5:**
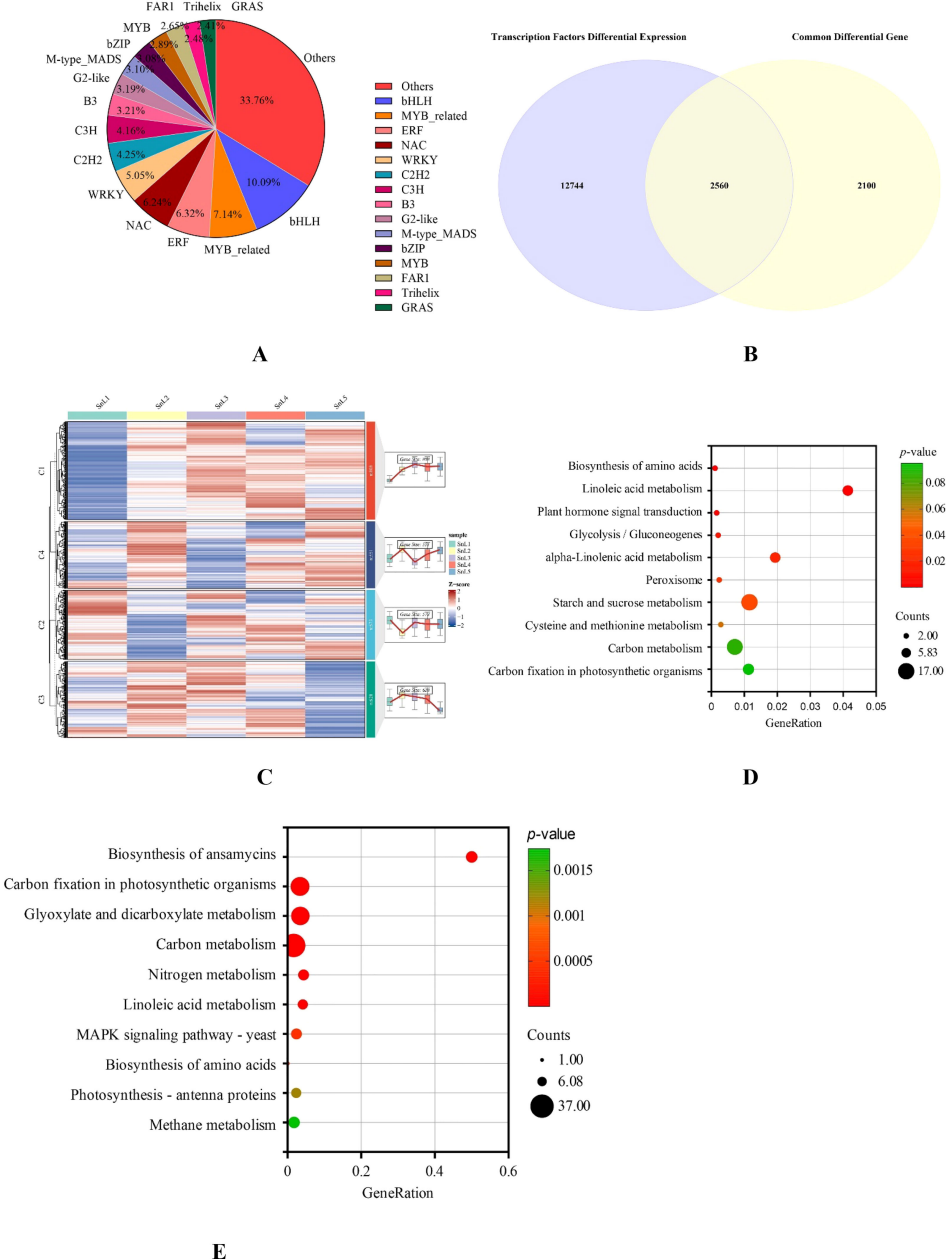
Analyzing the composition and functionality of a group of transcription factors that are expressed differently. **(A)** Proportion of transcription factor families with differential expression; **(B)** intersection of differentially expressed transcription factors and common differential genes at different growth stages of *Solanum nigrum* leaves taken together. **(C)** Temporal order analysis of common differential transcription factors at different growth stages of *Solanum nigrum* leaves. **(D)** Analysis of the cluster 1 transcription factor’s KEGG pathway enrichment. **(E)** Analysis of the cluster 4 transcription factor’s KEGG pathway enrichment.

For transcription factor prediction, we submitted the sequences of the 30,311 DEGs that were screened to the PlantTFDB4.0 database (http://planttfdb.cbi.pku.edu.cn/). A total of 15,304 transcription factors were identified. These transcription factors have been classified into different families, including bHLH, MYB-related, ERF, NAC, WRKY, C2H2, and C3H (**Figure 5A**). Taking the intersection of the predicted transcription factors with the differential genes common to the five growth stages revealed 2560 transcription factors out of 4659 common differential genes (**Figure 5B**). To further recognize the functions of these 2560 transcription factors, we clustered the 2560 differentially expressed transcription factors into four clusters using the mfuzz clustering algorithm (**Figure 5C**). The expression of cluster 1 was lowest at the leaf bud stage, and its expression increased with the growth and development of *S. nigrum* leaves, reaching a maximum at the mid-leaf stage. It is worth noting that the expression of the cluster 4 gene appeared opposite to that of the cluster 1 gene; the expression of the cluster 4 gene was highest at the leaflet stage, and its expression gradually decreased with the growth and development of *S. nigrum* leaves and reached the lowest at the maturity stage. Cluster 2 and Cluster 3 gene expression changes do not correspond to leaf margin changes in *S. nigrum*.

KEGG analysis of cluster 1 and cluster 4 genes revealed that cluster 1 genes were primarily associated with metabolic pathways such as amino acid biosynthesis, linoleic acid metabolism, and phytohormone signaling (**Figure 5D**). Cluster 4 genes were primarily associated with metabolic pathways such as ansamycin biosynthesis, carbon fixation in photosynthetic organisms, glyoxylate and dicarboxylic acid metabolism, and carbon metabolism (**Figure 5E**). These results suggest that transcription factors may be influencing plant growth and differentiation by regulating signal transduction and sugar synthesis.

### 3.6 Module-phenotype association analysis based on WGCNA

**Figure 6:**
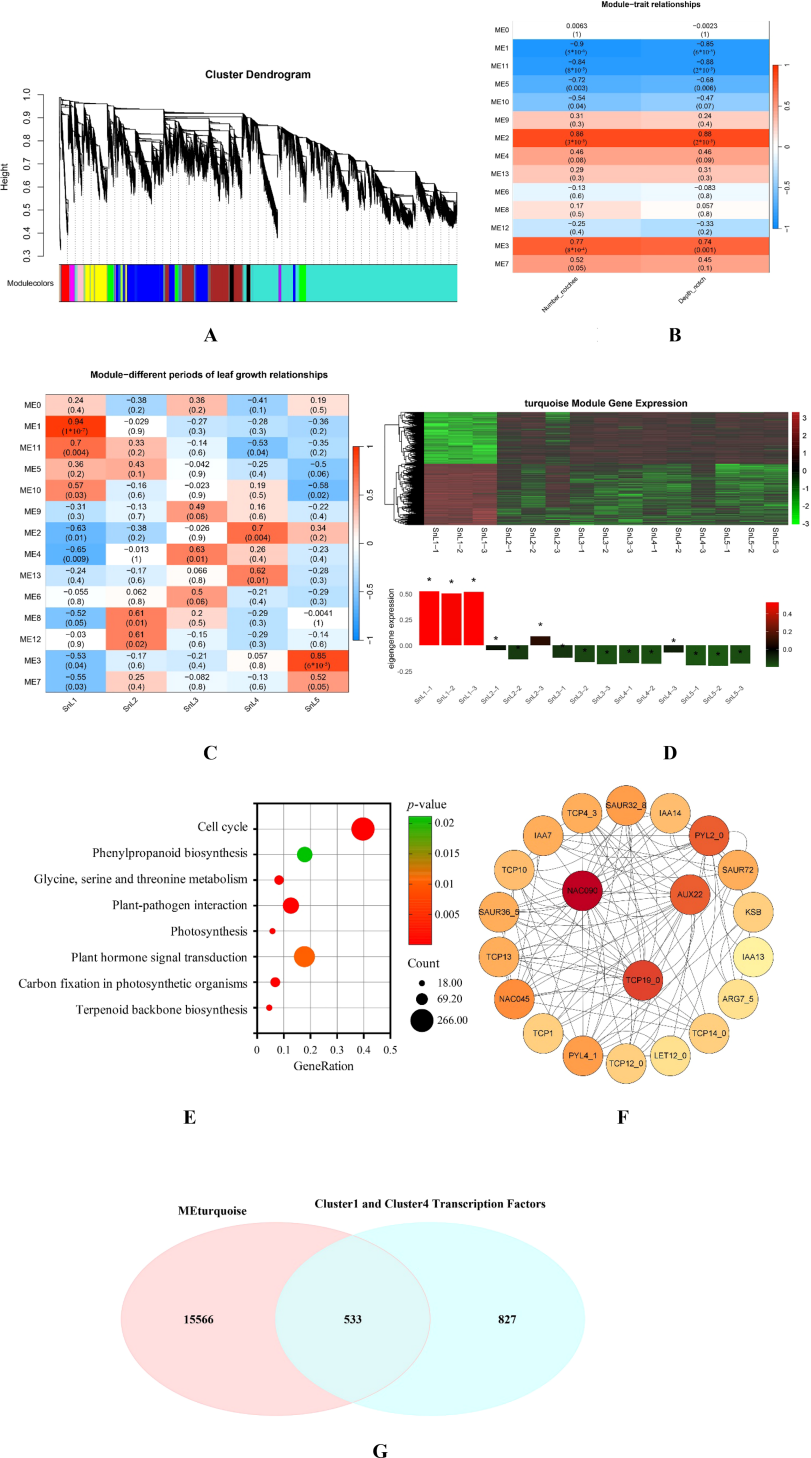
Co-expression network modules and pivotal transcription factors regulating leaf margin fission in *Solanum nigrum*. **(A)** Layered clustering tree diagram of weighted gene co-expression network analysis (WGCNA) module; color denotes different co-expression modules (β soft threshold power = 6, scale-free topology model suitable for R^2^ > 0.8); **(B)** heatmap of the relationship between Module 1 and leaf margin fission traits shows the relationship between Module 1 and the number of notches and notch depth of the leaf; negative correlations are shown in blue and positive correlations are shown in red. **(C)** A heatmap of Module 1 in relation to different growth stages of *Solanum nigrum* leaves shows the relationship between Module 1 and the different growth stages of the leaves. **(D)** Expression trends of turquoise module (ME1) genes at different growth stages of *Solanum nigrum* leaves (leaf buds, small leaves, medium leaves, large leaves, mature leaves). **(E)** KEGG analysis of Module 1: The figure shows 9 pathways associated with plant leaf differentiation. **(F)** Hub gene interaction networks: Weighted linkage of differential genes in leaf differentiation-related pathways (color shade reflects connectivity). **(G)** The turquoise module (ME1) genes and the cluster 1 and cluster 4 transcription factors intersect. The symbol * indicates the significance level of turquoise module (ME1) genes across different developmental stages of *Solanum nigrum* leaves. The greater the distance from the horizontal axis, the higher the significance level.

Using WGCNA, we established a co-expression network of 30,311 differentially expressed genes in *S. nigrum* from leaf buds to mature leaves to analyze the gene regulation network of leaf margin fission in this plant (**Figure 6A**). Correlations of the modules with different growth stages and leaf margin lobe traits (number of leaf notches and depth of notches) of *S. nigrum* leaves were calculated in **Figures 6B, C**, respectively. In the correlation analysis between modules and leaf phenotypes of *S. nigrum*, the modules with |r|>0.8 were ME1, ME2, and ME11. Considering that gene transcription and translation occur in a spatially and temporally sequential manner, the leaf bud stage (SnL1), during which the *S. nigrum* leaves are not yet fully differentiated, was selected for correlation analysis. However, in the SnL1 phase, ME1 was the only module exhibiting |r| > 0.8. This module was positively correlated with SnL1 and negatively correlated with the number and depth of notches in *S. nigrum* leaves. The above findings indicate that the differential gene expression profile of this module is consistent with the variation in leaf margin phenotypes in *S. nigrum*, and the number of differentially expressed genes clustered into this module was 16,099. Analysis of the gene expression patterns of all the modules revealed that the gene expression of the turquoise module (ME1) gradually declined with the growth and development of *S. nigrum* leaves and began to level off at the macrophyte stage (the stage of leaf morphogenesis) (**Figure 6D**). Therefore, it was hypothesized that the turquoise module (ME1) may regulate the formation of leaf margin lobes in *S. nigrum* and was selected for further analysis.

The KEGG analysis of the module 1 gene (**Figure 6E**) showed 9 pathways associated with plant leaf differentiation, all with *p*-values less than 0.01. Differentially expressed genes enriched in the pathway were subjected to network interaction analysis using the STRING database, and the results were visualized using Cytoscape (**Figure 6F**). The three most highly connected genes, *NAC90*, *TCP19_0*, and *AUX22*, were taken into focus. And from this reciprocal network diagram, we found that *NAC90* has an interaction link with the *TCP4* transcription factor. Previous studies have reported that *TCP4* can form dimers with *CUC2* and *CUC3*, respectively, thereby preventing the formation of CUC2-CUC3 dimers and leading to a reduction in the degree of leaf dentition/dissection. It is hypothesized that *NAC90* may influence the formation of leaf margin fission in plants by affecting the expression of the *TCP4* gene. Intersecting the genes in the turquoise module (ME1) with the transcription factors in clusters 1 and 4 in Conclusion 3.5 revealed that there are 533 shared differential transcription factors in the turquoise module (ME1). However, among the three key genes, only *SnNAC90* belongs to these 533 common differential transcription factors. In summary, *SnNAC90* has an important role in the regulation of leaf margin fission in *S. nigrum*; thus, *SnNAC90* will be the focus of the study for the next step of functional validation.

### 3.7 Effect of overexpression of the SnNAC90 gene on leaf margins of tobacco

**Figure 7:**
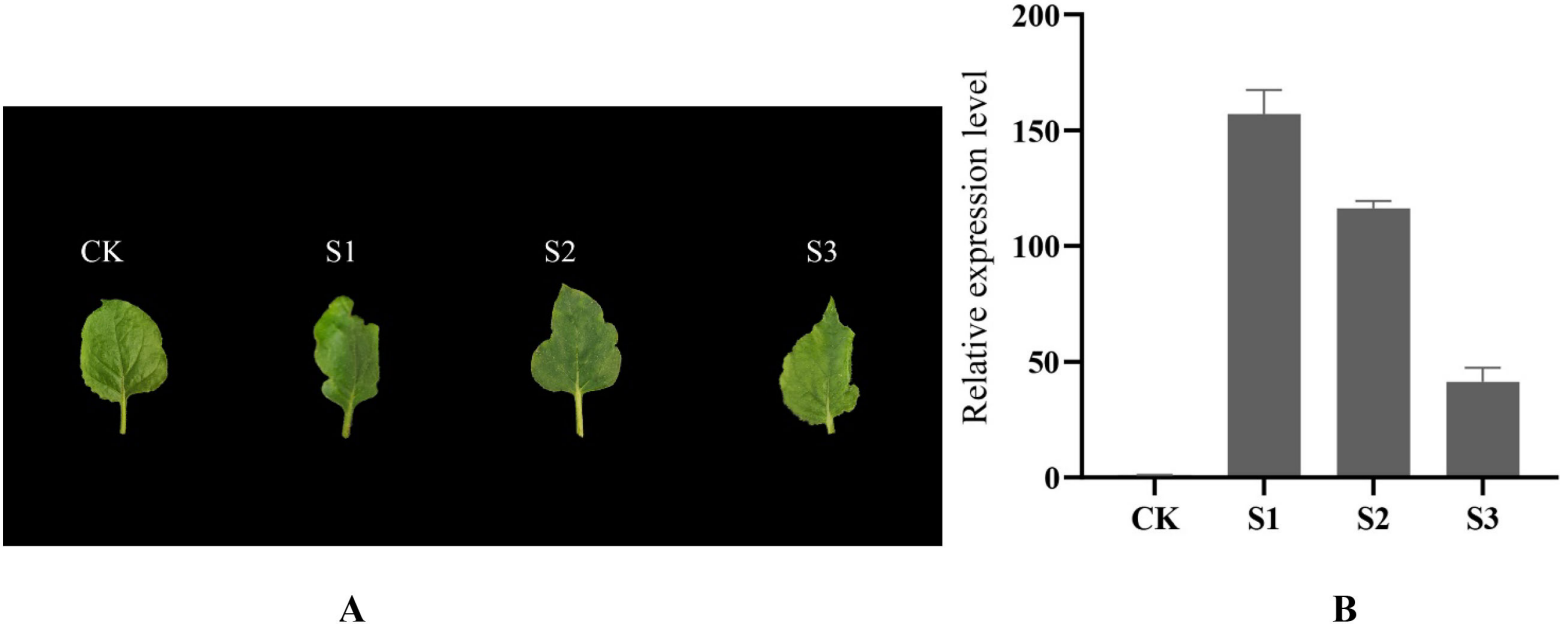
Effect of overexpression of *SnNAC90* on tobacco leaves. **(A)** Observation of leaf shape in tobacco overexpressing *SnNAC90*, CK as wild-type control; **(B)** In the qRT-PCR validation of transgenic tobacco, the transgenic strains at various expression levels are shown on the X-axis, while the Y-axis represents the relative expression levels. Tukey’s test and one-way ANOVA were the foundations of the approach (*p* < 0.01). The standard deviations of the technical replicates are referred to as error bars.

In tobacco, the *SnNAC90* gene was effectively expressed, and three genetic lines emerged. The *SnNAC90* gene was successfully incorporated into the tobacco genome, according to the results based on the relative expression levels of the gene using wild-type tobacco as a control. In **Figure 7A**, we observe that all three transgenic lines exhibit varying degrees of leaf margin dissection compared to the control (CK), with the most pronounced dissection observed in the S1 transgenic line. The S2 transgenic line shows less dissection than S1 but more than S3, exhibiting an intermediate level of dissection. The S3 transgenic line displays the least dissection, with a less pronounced phenotype. Based on qPCR quantitative analysis results (**Figure 7B**), the expression level of the SnNAC90 gene was significantly higher than that in other transgenic lines, exhibiting the most pronounced phenotype. In the S2 and S3 transgenic lines, SnNAC90 gene expression also showed a positive correlation with the phenotype. The aforementioned findings imply that variations in *SnNAC90* gene expression across these genetically modified lines could result in disparities in the extent of fission at tobacco leaf margins.

## 4 Discussion

Leaf margin fission in plants is an important phenotype for plants suited to environmental change. In herbaceous plants, deeply notched leaf plants are taller than shallowly notched leaf plants; the notching of the leaf margins gives the plant a greater advantage in its ability to grow longitudinally, and deeply lobed leaf plants capture light energy more quickly and compete better for limited light sources (Givnish and Vermeij, 1976). Leaf margin fission regulation mechanisms are being translated into agrotechnological innovations. Gupta et al. edited the tomato SlCUC gene by CRISPR/Cas9 to produce a strain with increased lobe depth, which showed a certain degree of yield per leaf area (Gupta et al., 2021). It has been shown that optimizing plant leaf lobe conformation can allow for an increase in canopy light energy utilization. In breeding for stress tolerance, the transfer of the *SoCUC4* gene of endive in rice allowed rice to improve survival under water stress (Wang et al., 2025). Studies on soil improvement, ecological management, and medicinal use of *S. nigrum* have been reported, and studies on the mechanism of leaf margin fission in *S. nigrum* have not been reported. In this study, we focused on the molecular regulatory mechanisms of leaf cleavage and smooth leaf phenotypes in *S. nigrum* and systematically analyzed the key regulatory networks of leaf cleavage development by integrating transcriptome sequencing, WGCNA, and overexpression transgene technology.

With the advancement of high-throughput sequencing methods, transcriptome sequencing has also become one of the means of mining plant phenotypes, which is used to study the biological functions and regulation of relevant genes in the plant body by determining the expression of the genes in different tissues, conditions, or time points. With the increasing sophistication of weighted gene co-expression network analysis (WGCNA), WGCNA was widely used in various aspects of plants (Langfelder and Horvath, 2008; Meng et al., 2022; Wu et al., 2022; Li et al., 2025). In this study, for the first time, the gene co-expression network was constructed based on WGCNA dynamically associated gene modules related to leaf margin fission with different growth stages of *S. nigrum* leaves. Given that gene transcription and translation occur in spatially and temporally sequential manner, the leaf bud stage (SnL1), during which the *S. nigrum* leaves are not yet fully differentiated, was selected for correlation analysis. WGCNA analysis revealed that the turquoise module (ME1) was highly positively correlated with the leaf bud growth stage and negatively correlated with both the number and depth of notches in *S. nigrum* leaves. This finding is consistent with the observed changes in leaf margin during leaf development. Protein interaction analysis of ME1 identified three key genes: NAC90, TCP19_0, and AUX22. Among them, the *AUX22* gene is involved in the limiting transport of phytochemicals, and *TCP4* can form dimers with *CUC2* and *CUC3*, respectively, thus preventing the formation of the CUC2-CUC3 dimer and leading to a reduction in leaf abscission/division. In summary, *NAC90*, *TCP19_0*, *TCP4* and *AUX22* were identified as key focuses for later studies.

NAC (NAM/ATAF/CUC) transcription factors are a family of plant-specific regulatory proteins that play a central role in leaf morphogenesis, especially leaf lobing, by coordinating cell division, differentiation, and boundary tissue development (Lelandais-Briere et al., 2010). Typical NAC proteins contain a conserved DNA-binding domain at the N-terminus and a highly variable transcriptional regulatory domain at the C-terminus, which regulates the spatiotemporal expression pattern of downstream genes by binding to specific cis-elements (e.g., CATGTG motifs) in the promoters of target genes (Kim et al., 2007; Hao et al., 2010; Shao et al., 2015; Singh et al., 2021). It has been shown that members of the CUC (CUP-SHAPED COTYLEDON) subfamily (e.g., *CUC2*, *CUC3*) inhibit the expression of the growth hormone (IAA) polar transport protein PIN1 by suppressing the growth hormone (IAA) polar transport protein PIN1 in the leaf primordium, establishing a region of low growth hormone concentration that drives the formation of leaf margin serrations or lobes (Gao et al., 2008; Larsson et al., 2012). For example, the *Arabidopsis thaliana CUC2* mutant (*CUC2*-1) exhibits leaf margin smoothing, whereas *CUC2* overexpressing lines develop a deeply lobed or compound leaf phenotype, confirming its dose-dependent regulation of leaf lobe development (Nikovics et al., 2006; Li et al., 2020; Wen et al., 2022). In addition, CUC proteins act synergistically with KNOX family transcription factors to promote cell proliferation in the lobular meristematic tissue region by activating the cell cycle protein gene *CYCB1*, while repressing differentiation-related genes to maintain the undifferentiated state, thereby finely regulating the depth and number of lobular cleavage (Koyama et al., 2010; Spinelli et al., 2011; Komaki and Sugimoto, 2012; Blein et al., 2013). Only *CUC2* and *CUC3* of the NAC transcription factor family have been found to affect plant leaf margin fission. For the focus of this study, *NAC90* was mainly concentrated on plant resistance. For example, overexpression of the *NAC90* gene in *Arabidopsis thaliana* decreases NHP and SA synthesis, which attenuates plant leaf senescence and enhances plant disease resistance (Cai et al., 2024). Jia et al. found in *Phalaris arundinacea* that *NAC90* gene expression was elevated under salt stress treatment (Jia et al., 2023). In this study, the screened *SnNAC90* differential gene was transferred into tobacco by Agrobacterium tumefaciens, and tobacco overexpressing the *SnNAC90* gene showed different degrees of leaf cleavage traits. This result demonstrates that the *SnNAC90* gene may be involved in the regulation of leaf margin fission in *Solanum nigrum* and is exerting a positive regulatory role. Moreover, different transgenic strains showed different degrees of fission traits; combined with the results of qPCR analysis, this may be due to the differences in gene copy number between transgenic strains. The above findings provide some reference basis for plant seed resource innovation. Although some studies have reported that the *TCP4* transcriptional factor interacts with NAC transcription factors and thus affects the leaf margin division of plant leaves, the interaction between *SnNAC90* and *SnTCP4* was not experimentally verified in this study, which is a shortcoming of the study.

## Data Availability

The original contributions presented in the study are included in the article/**Supplementary Material**. Further inquiries can be directed to the corresponding authors.
