# Supplementary figures and images for: The molecular mechanism of leaf margin fission in Solanum nigrum revealed by combined PPI network and WGCNA and functional validation of SnNAC90
Source: Front Plant Sci. 2025 Nov 18;16:1706416. doi: 10.3389/fpls.2025.1706416 (PMC12673809; doi:10.3389/fpls.2025.1706416)

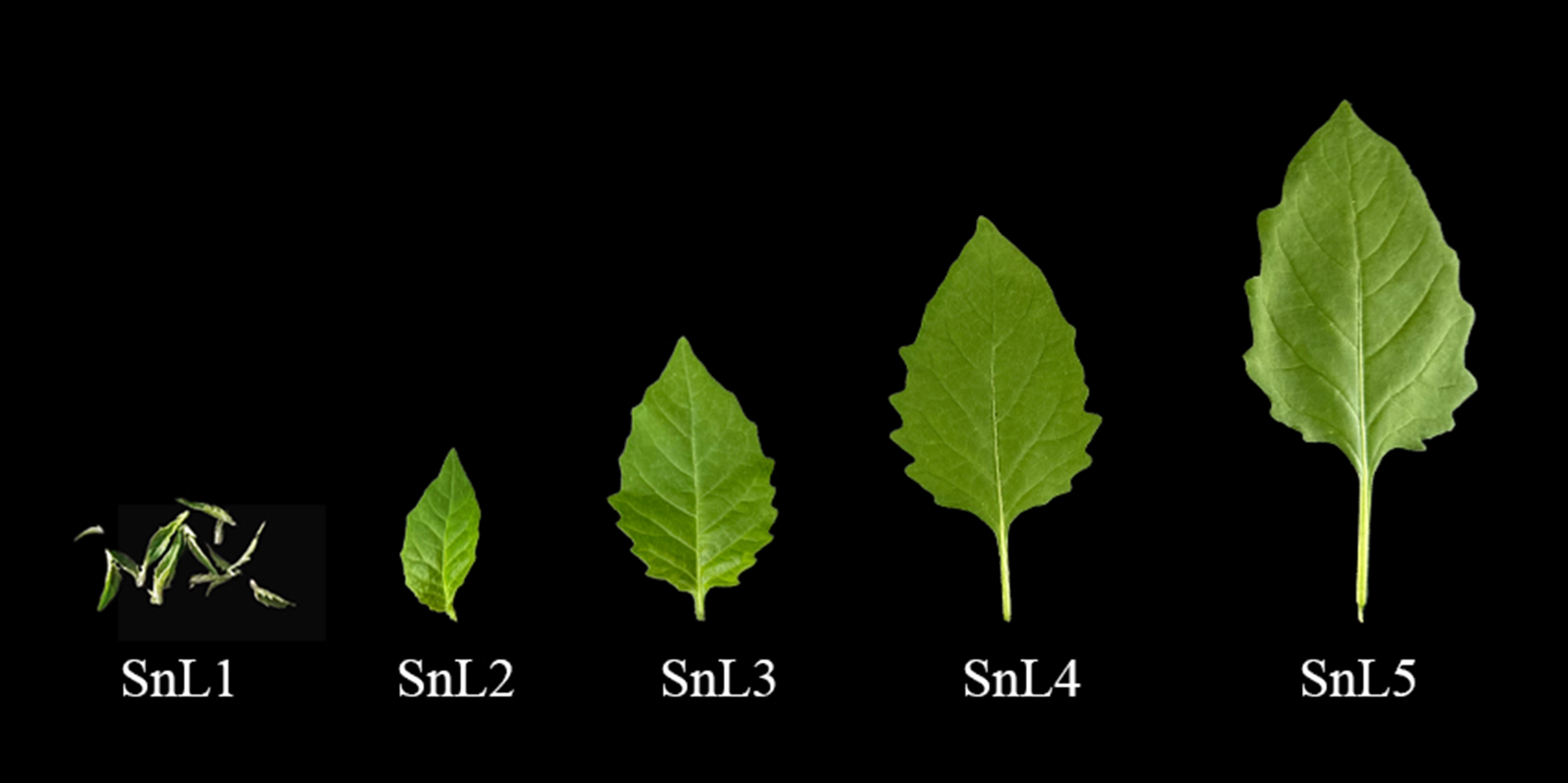

Supplement: Supplementary file 1 [file DataSheet1.zip › Supplementary Materials/Figure S1 sequencing samples.jpg]

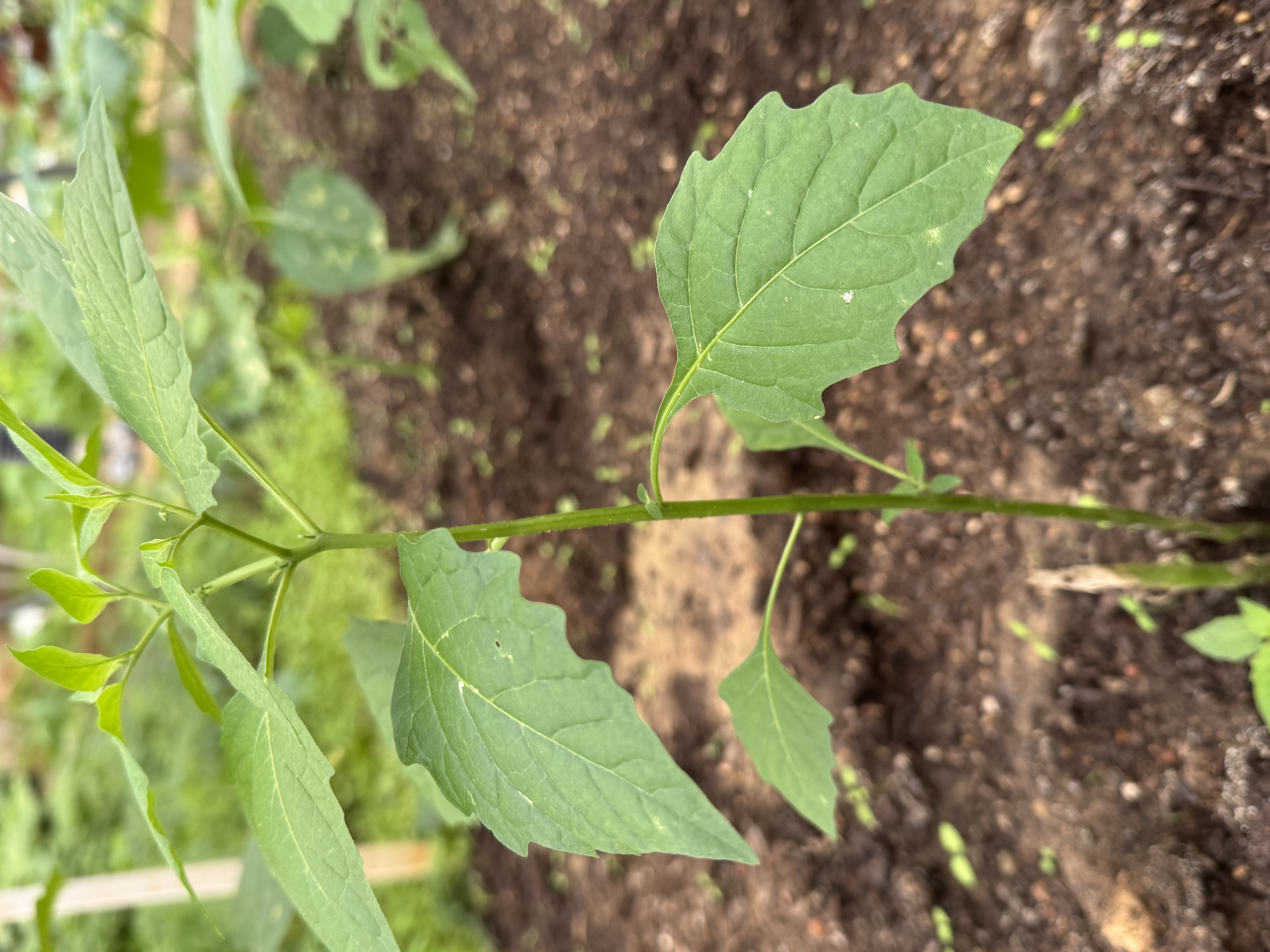

Supplement: Supplementary file 1 [file DataSheet1.zip › Supplementary Materials/Figure S2Solanum nigrum seedling.jpg]

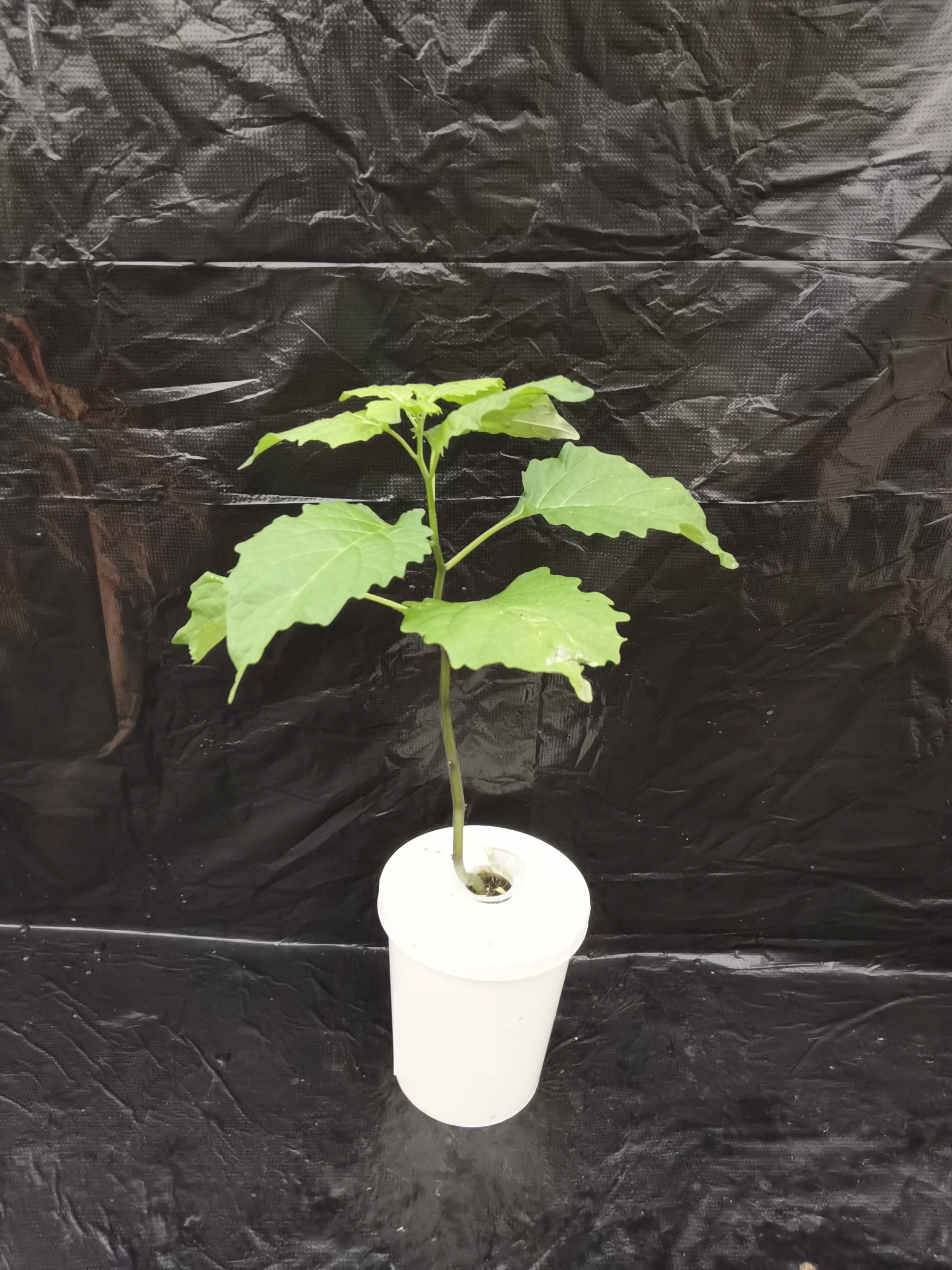

Supplement: Supplementary file 1 [file DataSheet1.zip › Supplementary Materials/Figure S3Solanum nigrum seedling.jpg]
